# Label-free colorimetric detection of mercury via Hg^2+^ ions-accelerated structural transformation of nanoscale metal-oxo clusters

**DOI:** 10.1038/srep16316

**Published:** 2015-11-12

**Authors:** Kun Chen, Shan She, Jiangwei Zhang, Aruuhan Bayaguud, Yongge Wei

**Affiliations:** 1 Department of Chemistry, Tsinghua University, Beijing 100084, P. R. China; 2 State Key Laboratory of Natural and Biomimetic Drugs, Peking University, Beijing 100191, P. R. China

## Abstract

Mercury and its compounds are known to be extremely toxic but widely distributed in environment. Although many works have been reported to efficiently detect mercury, development of simple and convenient sensors is still longed for quick analyzing mercury in water. In this work, a nanoscale metal-oxo cluster, (*n*-Bu_4_N)_2_[Mo_5_NaO_13_(OCH_3_)_4_(NO)], (MLPOM), organically-derivatized from monolacunary Lindqvist-type polyoxomolybdate, is found to specifically react with Hg^2+^ in methanol/water via structural transformation. The MLPOM methanol solution displays a color change from purple to brown within seconds after being mixed with an aqueous solution containing Hg^2+^. By comparing the structure of polyoxomolybdate before and after reaction, the color change is revealed to be the essentially structural transformation of MLPOM accelerated by Hg^2+^. Based on this discovery, MLPOM could be utilized as a colorimetric sensor to sense the existence of Hg^2+^, and a simple and label-free method is developed to selectively detect aqueous Hg^2+^. Furthermore, the colorimetric sensor has been applied to indicating mercury contamination in industrial sewage.

Mercury has shown an extensive range of poisonous actions to human, and the most susceptible target organ is brain. Exposure to mercury or its compounds during pregnancy and early childhood can cause strong damages to the central nervous system, such as neurodevelopmental disorders and subclinical brain dyfunction^1^. Developmental neurotoxicity will lead to various cognitive and motor disorders in the later life span^2^,^3^. The consumption of fish and seafood is a major absorption source of mercury in humans^4^, and mercury would constantly enter aquatic systems through anthropogenic emissions over time. Mercury detection in water is of great significance due to its wide distribution in environment and bioaccumulation through the food chain.

Mercury monitoring in environmental water has gained significant attention in recent years. There are considerable efforts dedicated to the development of selective and sensitive mercury detection methods. Among them, fluorescent and colorimetric sensors are the most convenient techniques in monitoring the level of mercury^5^,^6^. Because of its closed-shell d^10^ configuration, mercury(II) ion itself has no optical spectroscopic signature, which limits the kinds of methods that can be applied to its on-field or *in vivo* study. The use of mercury-responsive sensor that provides immediate optical feedback is arguably best suited for monitoring Hg^2+^ in either environmental or biological contexts.

The optical feedback, in the form of changes in solution fluorescence or UV–vis absorption, is resulted from a Hg^2+^-induced perturbation of an optical probe. In the past decades, a variety of remarkable probes based on small organic molecules^7^,^8^, polymeric materials^9^, biomolecules^10^,^11^, hydrogels^12^, quantum dots^13^, gold nanoparticles^14^,^15^,^16^,^17^, carbon nanoparticles^18^, and graphene^19^,^20^ have been developed for optical Hg^2+^ detection in drinking water^13^, environmental water^16^,^21^ and in cells or organisms^8^,^18^,^20^ to monitoring distribution of mercury. To meet the criteria for on-field analysis or rapid screening, colorimetric detection is particularly attractive for point-of-use applications due to its simplicity and can provide readout by the naked eye without any affiliated apparatus assistant^6^. Design of next generation of colorimetric sensors are demanded to have these advanced properties: low-cost, quick response, practical applicability and easy preparation. Organic chromophoric probes often have critical limitation of poor solubility in aqueous sample detection, and they often require complicated, multistep probe preparation and/or sophisticated organic synthesis techniques. Nanoparticles composed of noble metals, such as gold nanoparticles^14^,^15^,^16^,^17^ and silver nanoparticles^21^, on one hand are much expensive in high cost of materials. On the other hand, in most case these nanoparticles need post-modification^14^,^15^,^16^,^17^ to tether a mercury chelating or binding moiety to function as a sensor. This post-modification increases the specificity to Hg^2+^, but it is cost- and time-consuming. In contrast, a label-free approach is simple and rapid, without any step of modification or labeling^10^.

To develop a low-cost, simple and selective method for Hg^2+^ detection, polyoxometalates (POMs) are introduced into the optical sensing systems as colorimetric sensors. POMs are a group of intriguing materials of metal-oxo clusters with nano-sized molecular geometry which consist of transition metal oxyanions with unique optical, electronic and magnetic properties^22^,^23^,^24^. Structural diversity makes POMs and their derivatives found numerous applications mainly in catalysis^25^,^26^,^27^,^28^, medicine^29^,^30^ materials science^31^,^32^,^33^,^34^,^35^, molecular electronics^36^, and energy storage^37^,^38^. Although the influential “molybdenum blue test” has been applied as a simple, fast, and sensitive method by generations of chemists for almost 200 years^39^, POM-based sensors still comprise a minority in analytical chemistry. Recently, POMs have been proved to show oxidase or peroxidase mimetic activities^40^, which could catalyze 3,3′,5,5′-tetramethylbenzidine (TMB) to its oxidized form with a blue color to indirectly determine folate-expressing cancer cells in colorimetric assay. However, POM-based archetypes as colorimetric sensors for direct label-free sensing of heavy metal ions are extremely rare. Different structure types may invest POMs different optical absorption activities. Structure transformation will likely cause color variation of the POM solution. In this paper, based on the color change stimulated by Hg^2+^, we report that a nanoscale metal-oxo cluster, the organically-derivatized monolacunary Lindqvist-type polyoxomolybdate^41^, (*n*-Bu_4_N)_2_[Mo_5_NaO_13_(OCH_3_)_4_(NO)], MLPOM (Fig. 1), can be used to directly colorimetric detect mercury ions in aqueous solution (Fig. 2).

## Results

### Structural transformation driven by Hg(II)

**Figure 2:**
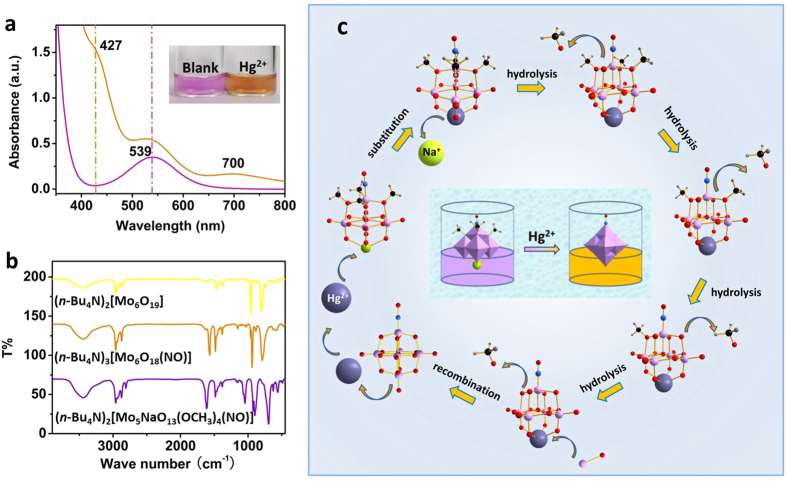
Colorimetric determination of Hg(II) by MLPOM. (**a**) UV-vis spectra of MLPOM (50 μM) in the absence (purple) and presence (brown) of Hg^2+^ ions (10 μM). Inset: photographs of MLPOM (50 μM) in the absence (purple) and presence (brown) of Hg^2+^ ions (10 μM). (**b**) IR spectra of (*n*-NBu_4_)_2_[Mo_5_NaO_13_(OCH_3_)_4_(NO)] (purple), (*n*-Bu_4_N)_3_[Mo_6_O_18_(NO)] (brown) and (*n*-Bu_4_N)_2_[Mo_6_O_19_] (yellow). (**c**) Schematic diagram of the probable structural transformation procedure from [Mo_5_NaO_13_(OCH_3_)_4_(NO)]^2−^ to [Mo_6_O_18_(NO)]^3−^ in the presence of Hg^2+^. Ball-and-stick representation of [Mo_5_NaO_13_(OCH_3_)_4_(NO)]^2−^, [Mo_6_O_18_(NO)]^3−^ and their possible intermediates: Mo, light purple; Na, lime; O, red; N, blue; C, black; H, light gray; Hg, blue gray.

In this report, MLPOM·2CH_3_OH was synthesized according to the reported methanol-mediated method^41^. When dissolved in methanol, the solution of MLPOM was purple. However, as observed, by dropping Hg^2+^ aqueous solution into the system, the solution color changed into brown in a few seconds (Fig. 2a inset). In order to confirm the substance in the brown solution, the solvent was evaporated slowly and dark brown block crystals were obtained. Interestingly, the UV-vis (Fig. 2a) and IR (Fig. 2b) spectra of these brown crystals were consistent with the previously reported saturated Lindqvist-type structure, [Mo_6_O_18_(NO)]^3−^, of which one terminal oxygen atoms is substituted by a nitrosyl group^41^,^42^. Moreover, electrospray ionization mass spectrometry (ESI-MS) and the single X-ray diffraction further verified the dark brown crystals to be (*n*-NBu_4_)_3_[Mo_6_O_18_(NO)] (See Supplementary Fig. S1f). These results indicate that the color change of MLPOM is caused by the interaction of Hg^2+^ with MLPOM.

Being different from other nanoparticles, POMs have precisely defined chemical structures, size, shape symmetry, and surface functional groups. The MLPOM is a methanol-derivatized lacunar Lindqvist-type polyoxomolybdate with size of *ca* 1 nm, which was first reported by Proust *et al.* in 1989^41^. Its [Mo_5_O_13_(OCH_3_)_4_(NO)]^3-^ unit is in fact a multi-faced ligand that offers several coordination sites and displays various coordination modes^43^. The reaction of transition-metal species towards the single oxo-nitrosyl complex MLPOM could result in diverse coordination modes and give transition metal substituted POMs. In addition, it may act as a source of {MoO}^4+^ and {Mo(NO)}^3+^ units under appropriate conditions^44^.

It was reported that [Mo_5_NaO_13_(OCH_3_)_4_(NO)]^2−^ can spontaneously transform into [Mo_6_O_18_(NO)]^3−^ when it is dissolved in dichloromethane or acetonitrile (See Supplementary Fig. S2)^41^. However, according to our test, this transformation of MLPOM can be accelerated by Hg^2+^ and our finding might be referred to as a “Lewis acid-catalyzed” reaction. In mercury, relativistic effects in the valence shell of the elements reach a maximum and determine its reactivity to transition metals and their derivatives^45^. Mercury salts have been proved to be excellent catalysts for various transformations^46^. Meanwhile, Hg^2+^ is a typical soft Lewis acid, and the monolacunary Lindqvist-type MLPOM anion could be regarded as a Lewis base. MLPOM contains a [Mo_5_O_13_(OCH_3_)_4_(NO)]^3−^ unit acting as a multi-faced ligand^43^ that offers several coordination sites to bind mercuric ion. Upon the addition of Hg^2+^, the [Mo_5_O_13_(OCH_3_)_4_(NO)]^3−^ unit of MLPOM will release its Na^+^ and coordinate with Hg^2+^ to form a mercury substituted POM. Through carefully analyzing several ESI-MS spectra of MLPOM methanol solution with immediate addition of Hg^2+^ aqueous solution, probable peaks of Hg-Mo_5_ anionic fragments are casually caught, including [Mo_5_HgO_13_(OCH_3_)_4_(NO)]^−^, [Mo_5_HgO_14_(OCH_3_)_3_(NO)]^2−^, [Mo_5_HgO_15_(OCH_3_)_2_(NO)]^3−^, [Mo_5_HgO_16_(OCH_3_)(NO)]^4−^, and [Mo_5_HgO_17_(NO)]^5−^ (See Supplementary Fig. S1). The signals of these intermediates have fleeting life spans, indicating that they are rather unstable and quickly convert into other stable structures. The probably initial intermediate product is [Mo_5_HgO_13_(OCH_3_)_4_(NO)]^−^. Soon it is hydrolyzed by water and gradually loses its methoxyl groups. The possible final hydrolysis product is [Mo_5_HgO_17_(NO)]^5−^ which finally recombines with a {MoO}^4+^ unit to form [Mo_6_O_18_(NO)]^3−^ and releases one Hg^2+^ to enter another reaction cycle (Fig. 2c). MLPOM is practically insoluble in water (See Supplementary Fig. S3). Hydrolysis in water is imperceptible and slow unless being heated (See Supplementary Fig. S4). By heating at 80 °C for 30 min, yellow hydrolysis product is generated and characterized to be (*n*-NBu_4_)_2_[Mo_6_O_19_] by IR. Herein, under room temperature, structure transformation of MLPOM is triggered and accelerated dominantly by Hg^2+^.

This mercury detection method bears an uncanny resemblance to the famous Hach method to detect Si in water but with significant difference. The Hach method, namely the “molybdenum blue test”, is based on formation and reduction of Keggin-type polyoxometalates. Generally, silica and phosphate in the sample react with molybdate ion under acidic conditions to form yellow silicomolybdic acid and phosphomolybdic acid complexes. Addition of citric acid destroys the phosphate complexes. An amino acid is then added to reduce the yellow silicomolybdic acid to an intense blue color, which is proportional to the silica concentration. The similarity between our method and Hach method is the formation of a new polyoxometalate structure stimulated by analyte during assay. The difference is the optical read-out generated by alteration of POM molecular structure in our method versus by alteration of electronic structure in the molybdenum blue test.

Interestingly, after immersed in methanol, the crystal of (*n*-NBu_4_)_3_[Mo_6_O_18_(NO)] dissolves gradually while the solution becomes purple again, which indicates that (*n*-NBu_4_)_3_[Mo_6_O_18_(NO)] transforms back to MLPOM owing to the solvent effect (See Supplementary Fig. S5). This structure transformation in methanol was found reversible by introducing a small amount of reductant which can reduce Hg^2+^ to Hg^0^. The brown methanol solution of [Mo_6_O_18_(NO)]^3−^ was also gradually turned back to purple (See Supplementary Fig. S5) upon addition of a small amount of hydroxylamine hydrochloride. The reversible color change illustrated that the structure of [Mo_6_O_18_(NO)]^3−^ transformed back to monolacunary Lindqvist-type MLPOM when Hg^2+^ was reduced to Hg^0^. These results confirm that in this reaction system, the structure transformation of MLPOM is caused by the addition of Hg^2+^. The interaction of Hg^2+^ and MLPOM results in the structural transformation of MLPOM, and therefore it is attractive for developing a label-free approach in Hg^2+^ detection.

### Colorimetric sensing of Hg(II)

**Figure 3:**
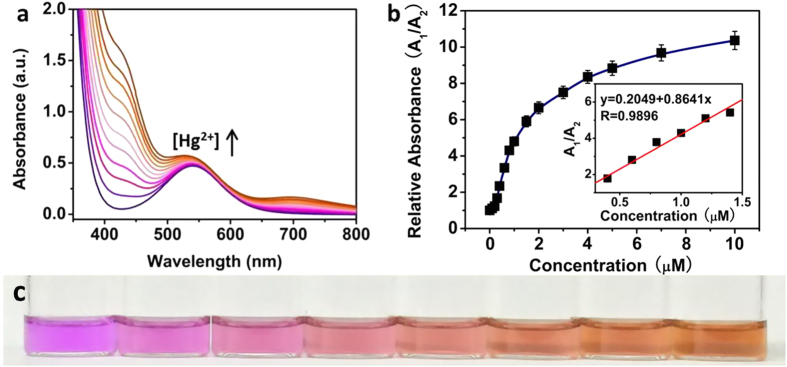
Measurements of Hg(II) in aqueous solution. (**a**) UV-vis spectra of MLPOM (50 μM) in the presence of different Hg^2+^ concentrations (from bottom to top: 0 μM, 0.25 μM, 0.5 μM, 0.8 μM, 1.2 μM, 1.5 μM, 2.0 μM, 3.0 μM, 5.0 μM, 7.0 μM, 10.0 μM). (**b**) Relative UV-vis absorbance (A_1_/A_2_, A_1_ is the absorbance of MLPOMs in methanol at 427 nm, A_2_ is the absorbance at 539 nm, respectively) of MLPOM (50 μM) as a function of Hg^2+^ concentration (0~10 μM). The inset shows the linear detection range for 0.2~1.4 μM of Hg^2+^. Error bars were calculated based on the standard deviation of three measurements. (**c**) Photographs of MLPOM in the presence of different Hg^2+^ concentration (from left to right: 0 μM, 0.05 μM, 0.25 μM, 0.6 μM, 0.8 μM, 1.0 μM, 1.5 μM, 2 μM).

The determination of Hg^2+^ in the environment in general, is key to assessing overall environmental and subsequent health impacts (e.g., accumulation and toxicity) of mercury species^4^. Relied on the transformation of MLPOM triggered by Hg^2+^, a simple method is developed for the detection of Hg^2+^. The original MLPOM exhibits a purple color in methanol. With the addition of Hg^2+^, the structure of MLPOM transformed immediately, resulting in a conversion of solution color from purple to brown (Fig. 2). As the concentration of Hg^2+^ increases, the solution color changes more obviously. The distinct color change shows MLPOM a potential colorimetric sensor in indicating the existence of Hg^2+^. To demonstrate the capability of MLPOM-based sensor for quantitative Hg^2+^ detection, UV-Vis was recorded to clarify the relationship between color conversion and the concentration of Hg^2+^ (Fig. 3a,b). In the UV-Vis spectra, MLPOM displays a weak band at 539 nm (Fig. 2a), which is characteristic of the [Mo_5_O_13_(OCH_3_)_4_(NO)]^3−^ unit and assigned to the d_xz_, d_yz_ → d_xy_ transition within the {Mo(NO)}^3+^ unit^41^,^42^,^43^. Each [Mo_6_O_18_(NO)]^3−^ also has one {Mo(NO)}^3+^ units, thus there is an absorbance at 539 nm but slightly stronger than MLPOM (Fig. 2a). Besides, a shoulder band at approximately 427 nm elevates as the concentration of Hg^2+^ increases, which is the characteristic transition of the π-type nonbonding terminal oxygen and nitrogen atoms to the molybdenum *d*-type LUMO.

Based on the intensity change of absorbance at 427 and 539 nm, the sensitivity and linear range of the assay were evaluated by investigating relative absorbance (A_1_/A_2_, A_1_ is the absorbance at 427 nm, A_2_ is the absorbance at 539 nm, respectively) of MLPOM in the presence of different concentrations of Hg^2+^ (0.05 ~ 10 μM). The color of MLPOM methanol solution gradually changed from purple to pink and at last brown as the concentration of Hg^2+^ increased (Fig. 3c). Spectral analysis proved that the relative absorbance of MLPOM was also enhanced with the increasing concentrations of Hg^2+^, and increased linearly over the Hg^2+^ concentration range of 0.2 ~ 1.4 μM (Fig. 3b). Figure 3c shows the typical solution colors in the presence of Hg^2+^ at series of gradient concentration. At a signal-to-noise ratio of 3, the limit of detection for Hg^2+^ was estimated to be 0.05 μM (10 ppb). Compared with the method based on DNA-functionalized gold nanoparticles^14^,^15^,^16^,^17^ for Hg^2+^ detection which is nontrivial as it typically involves careful rational design and substantial purification techniques, our sensors could achieve comparative sensitivity but are superior in low-cost and easier preparation.

To design colorimetric sensors for optical Hg^2+^ detection, these factors must be taken into account. First, containing a metal chelating or binding moiety to function as a sensor provides sufficient selectivity for Hg^2+^ over other components in the environmental or biological samples. [Mo_5_O_13_(OCH_3_)_4_(NO)]^3−^ unit in MLPOM can act as a multi-faced ligand^43^ to bind Hg^2+^, avoiding post-modification to tether a mercury chelating or binding moiety to the sensor. So MLPOM is a natural label-free probe to recognize Hg^2+^. Second, containing at least one optical element is capable of absorbing or emitting light. Mercury binding alters either the electronic structure or the molecular structure of the MLPOM-based sensor, leading to an alteration in the intensity or wavelength of light absorption which permits visualization of these changes. Third, a fast and readily detectable response to Hg^2+^ is also important for quick read-out use. Because of the extremely short-lived Hg-Mo_5_ anionic fragments, the response time of MLPOM-based sensor towards Hg^2+^ is within several seconds. Therefore, the MLPOM-based sensor is suitable for developing label-free method in quick Hg^2+^ assay.

### Selective sensing of Hg(II)

**Figure 4:**
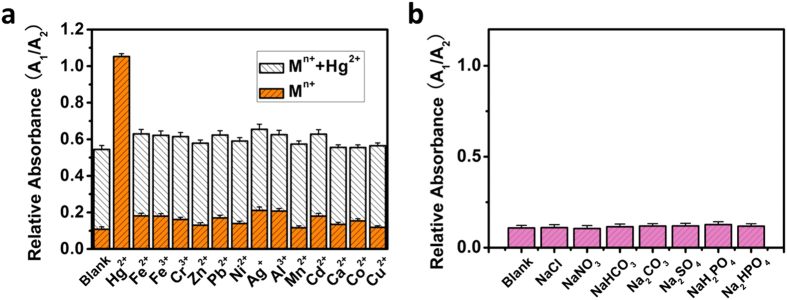
Selective sensing of Hg(II) by MLPOM. (**a**) Relative absorbance (A_1_/A_2_, A_1_ is the absorbance of MLPOMs in methanol at 427 nm, A_2_ is the absorbance at 539 nm, respectively) of MLPOM (50 μM) in the presence of different metal ions (M^n+^, orange columns, the concentration of Hg^2+^ ion was 0.8 μM, the concentration of other metal ions were 10 μM) or in the presence of different metal ions mixed with Hg^2+^ ion (M^n+^ + Hg^2+^, write columns, the concentration of Hg^2+^ ion was 0.5 μM, the concentrations of other metal ions were 5 μM). (**b**) Relative absorbance (A_1_/A_2_, A_1_ is the absorbance at 427 nm, A_2_ is the absorbance at 539 nm, respectively) of MLPOM (50 μM) in the presence of different anions (10 μM). Error bars were calculated based on the standard deviation of three measurements.

The selectivity of this assay for Hg^2+^ detection was examined by testing the response of MLPOM-based colorimetric sensors to other environmentally relevant metal ions. There are negligible variations on the UV-vis absorbance of MLPOM caused by 10 μM of Fe^2+^, Fe^3+^, Cr^3+^, Zn^2+^, Pb^2+^, Ni^2+^, Ag^+^, Al^3+^, Mn^2+^, Cd^2+^, Ca^2+^, Co^2+^ and Cu^2+^. However, MLPOM casted color changes as well when met a large amount of relevant metal ions in methanol solution. Generally, MLPOM showed 4~5 times stronger reactivity toward 0.8 μM Hg^2+^ than other metal ions even at the concentration of 10 μM which is beyond the linear range of MLPOM-based colorimetric Hg(II) sensor (Fig. 4a). Meanwhile, the mixtures of metal ions and Hg^2+^ were also tested using MLPOM-based colorimetric sensor. The relative absorbance of MLPOM was raised considerably in the case of addition of Hg^2+^ (Fig. 4a). These results indicate that the selectivity of POM-based colorimetric sensors can be as good as DNA-functionalized gold nanoparticles^14^,^15^,^16^,^17^ and also can be visualized with the naked eye. In addition, MLPOM exhibited robust performance to various anions, such as Cl^−^, NO_3_^−^, HCO_3_^−^, CO_3_^2−^, SO_4_^2−^, H_2_PO_4_^−^ and HPO_4_^2−^ (Fig. 4b). The capacity of resisting disturbance against other metal ions made MLPOM suitable for monitoring and measuring the content of Hg^2+^ discharged from industrial sewage.

### Colorimetric sensing of Hg(II) in industrial sewage

**Figure 5:**
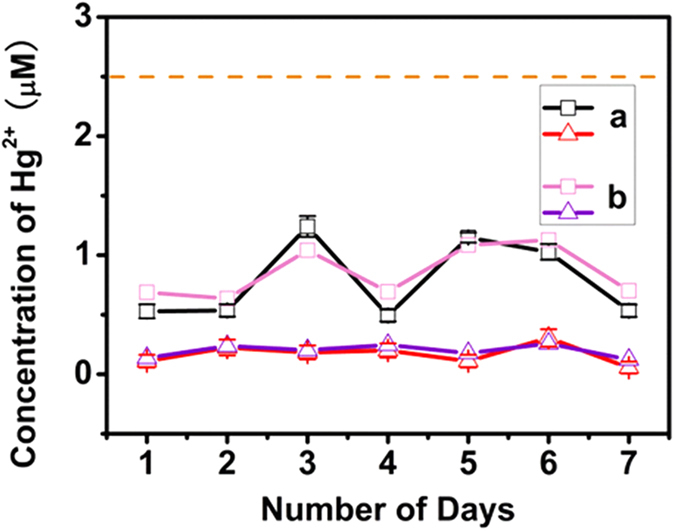
Concentration fluctuation of Hg^2+^ in industrial sewage. The samples were collected from electrolytic plant (□) and refining workshop (∆) over a period of one week. The concentration of Hg^2+^ was monitored by MLPOM-based sensors (**a**), blank line and red line) or atomic emission spectrometer (**b**), pink line and purple line). Orange dashed line was used to mark the emission standards of pollutants for mercury industries. Error bars were calculated based on the standard deviation of three measurements.

The feasibility of the proposed sensors for total mercury detection in industrial sewage was investigated by mixing disposed water samples with MLPOM methanol solution. The samples were collected and sealed in clean plastic bottles with tight-fitting caps. After transporting to laboratory, samples are analyzed immediately. The emission standards of pollutants for mercury industries^47^ were announced by the Ministry of Environmental Protection of the People’s Republic of China in May, 2014, in which mercury was limited to 0.05 mg/L (50 ppb) at the discharge outlets of production equipment. From the UV-vis absorbance of MLPOM, the total Hg^2+^ in each water sample was calculated to be less than 0.25 μM (50 ppb), below the national standard (Fig. 5). The test results were almost in accordance with those recorded by atomic fluorescence spectrometer, confirming the favorable ability of the POM-based sensors to rapidly estimate the Hg^2+^ concentration in waste water discharged from metal smelting enterprises.

## Discussion

Taking the advantage of structural transformation triggered by Hg^2+^, a method of label-free determining the existence and concentration of Hg^2+^ was constructed with the aid of an obvious color change by an organically-derivatized sodium-substituted monolacunary Lindqvist-type polyoxomolybdate with nano size. MLPOM was found to specifically react with Hg^2+^ in methanol/water, and rapidly transformed into the saturated nitrosoyl-substituted Lindqvist-type polyoxomolybdate. Spectra study indicated that this method could be applied to detect Hg^2+^ ion in aqueous media with remarkably high selectivity and amendable sensitivity. Compared with noble metal nanoparticles, POM-based colorimetric sensors are much cheap, easy-prepared, and can be expanded to bulk-production. Most importantly, they do not need ingenious design and substantial purification techniques, further demonstrating the feasibility of POMs in analytical applications. Considering the structure variation of POMs, this simple test system could be extended to other structure type for the rapid screening of metal ions.

## Methods

### Materials and measurement

Hydroxylamine hydrochloride was purchased from Shanghai Chemical Reagent Company (Shanghai, China). Bu_4_NBr was purchased from Beijing Chemical Industry Group Co., Ltd. (Beijing, China). (NH_4_)_6_Mo_7_O_24_·4H_2_O, dicyclohexylcarbodiimide (DCC), NaOH, anhydrous methanol, mercury perchlorate and other metal salts were purchased from Sinopharm Chemical Reagent Co., Ltd. (Beijing, China). All the reagents were of analytical grade and used without further purification. All aqueous solutions were prepared using ultrapure water (≥18 MΩ, Milli-Q, Millipore). The water samples were collected three times every day over a period of one week from electrolytic plant and refining workshop of one smelter in Daye, China. Infrared (IR) spectra were recorded on a FTIR spectrometer (Perkin–Elmer, USA). UV/Vis absorption spectra were recorded on a UV-2100S spectrometer (Shimadzu, Japan). Elemental analysis was measured with a ThermoQuest FLASH-1112 instrument (Thermo, USA). Total mercury in water samples were determined by LC-AFS 9800 atomic fluorescence spectrometer (Haiguang, China). All XRD data were collected on a Rigaku RAXIS-SPIDER IP diffractometer with graphite-monochromatized Moκ_α_ radiation (*λ* = 0.71073 Å) at 100 K. Data collection and reduction, cell refinement, and experiential absorption correction for all compounds were performed with the Rigaku RAPID AUTO software package (Rigaku, 1998, Version 2.30). The electrospray mass spectra (ESI-MS) were measured on a Finngan LCQ Deca XP Plus ion-trap mass spectrometer (San Jose, CA), and experiment was carried out in the negative-ion mode using methanol or CH_3_CN as solvent.

### Synthesis of MLPOM

The precursor (*n*-Bu_4_N)_4_[α-Mo_8_O_26_] was synthesized according to an improved literature method^48^ by the addition of a Bu_4_NBr aqueous solution to (NH_4_)_6_Mo_7_O_24_·4H_2_O aqueous solution, from which the product immediately precipitated. The precipitates were washed and dried before use. The products of (*n*-Bu_4_N)_4_[α-Mo_8_O_26_] were confirmed by IR spectroscopy. MLPOM was synthesized according to the reported methanol-mediated method^41^ with minor modifications. Typically, a mixture of (*n*-Bu_4_N)_4_[α-Mo_8_O_26_] (2.15 g, 1.0 mmol), NaOH (0.096 g, 2.4 mmol) and DCC (0.518 g, 2.4 mmol) in 10 mL methanol was mixed with hydroxylamine hydrochloride (0.167 g, 2.4 mmol). The suspension was refluxed for 4 h. After cooling down to room temperature, the reaction solution was filtered to remove yellow precipitates of (*n*-Bu_4_N)_2_[Mo_6_O_19_]. The filtrate was purple, and purple crystals were obtained about 12 h later. The yield for the crystalline product after crystallization (based on Mo) was 17%. Elemental analysis (calcd, found for (*n*-Bu_4_N)_2_[Mo_5_NaO_13_(OCH_3_)_4_(NO)]): C (32.01, 32.12), H (6.22, 6.34), N (3.11, 3.14). IR (KBr pellet): 2960(s) cm^−1^, 2874(m) cm^−1^, 1615(s) cm^−1^, 1483(m) cm^−1^, 1382(w) cm^−1^, 1071(m) cm^−1^, 1045(s) cm^−1^, 924(s) cm^−1^, 899(vs) cm^−1^, 696(vs) cm^−1^, 626(w) cm^−1^, 578(w) cm^−1^, 489(w) cm^−1^. Putative assignment for major peaks in ESI spectrum (m/z, in methanol): [M]^2−^ calcd. for [Mo_5_NaO_13_(OCH_3_)_4_(NO)]^2−^, 432.41; found, 432.26; [M]^−^ calcd. for {(*n*-Bu_4_N)[Mo_5_NaO_13_(OCH_3_)_4_(NO)]}^−^, 1107.2; found, 1107.80. Cell parameter (X-ray diffraction) of MLPOM: *a* = 17.664(2) Å, *b* = 17.652(4) Å, *c* = 20.6735(14) Å, *β* = 108.079(9)°; *V* = 6127.8(18) Å^3^. The thermo gravimetric analysis of MLPOM is tested from room temperature up to 500 °C at a heating rate of 10 °C/min under Ar atmosphere (See Supplementary Fig. S6). The first 1.38% weight loss at below 200 °C can be accounted for by solvent associated with the compound. The weight loss starting at 210 °C of 46.13% corresponds to the loss of the organic cations and the ligands (theoretically 47.34%). The results of thermo gravimetric analysis clearly demonstrate that the stability of MLPOM is not obviously affected below 210 °C. Hence, the driving force of heat is excluded from bringing in structure transformation of MLPOM at room temperature.

### Detection of aqueous mercury ions

A series of standard Hg^2+^ solutions were prepared by stepwise diluting stock solution following the instruction. In a normal detection procedure, 1 mL MLPOM methanol solution was mixed with Hg^2+^ aqueous solution of different concentrations (0~10 μM) under shaken. Unless otherwise specified, the MLPOM methanol solution was kept reaction with Hg^2+^ aqueous solution at room temperature for 20 sec. After 20 sec vigorously shaking, the mixture was piped into 1 cm quartz cuvette, and recorded the UV-vis absorption spectra. Each measurement was repeated three times. MLPOM is slightly soluble in ethanol (See Supplementary Fig. S2) and practically insoluble in water (See Supplementary Fig. S3). It could spontaneously transform into saturated Lindqvist-type polymolybdate^41^, (*n*-Bu_4_N)_3_[Mo_6_O_18_(NO)], in acetonitrile, and the structure transformation was also occurred in acetone, dimethylsulfoxide, *N, N*-dimethylacetamide, and *N, N*-dimethylformamide (See Supplementary Fig. S2). Owing to the solvent effect, all the test procedure was carried out in methanol/water solution. The structure of [Mo_6_O_18_(NO)]^3−^ was verified by ESI and the single X-ray diffraction. Putative assignment for major peaks in ESI spectrum (m/z, in acetonitrile): [M]^3−^ calcd. for [Mo_6_O_18_(NO)]^3−^, 297.88; found, 295.78; [M]^2−^ calcd. For {H[Mo_6_O_18_(NO)]}^2−^, 447.32; found, 445.67; [M]^2−^ calcd. for {(*n*-Bu_4_N)[Mo_6_O_18_(NO)]}^2−^, 568.05; found, 568.81; [M]^−^ calcd. for {(n-Bu_4_N)H[Mo_6_O_18_(NO)]}^−^, 1137.11; found, 1138.63; [M]^−^ calcd. for {(*n*-Bu_4_N)_2_[Mo_6_O_18_(NO)]}^−^, 1378.57; found, 1377.91. Cell parameter (X-ray diffraction) of [Mo_6_O_18_(NO)]^3−^: *a* = 23.9948(5) Å, *b* = 16.8568(4) Å, *c* = 16.6110(3) Å, *β* = 97.7229(19)°; *V* = 6657.8(2) Å^3^.

### Test of waste water samples

The water samples were under nitrification with sulfuric acid and 0.3 M potassium permanganate for 1 h to convert all the mercury species into mercuric ion (Hg^2+^) in aqueous media, and adjusted to ~pH 6.8 by addition of 0.1 M NaOH to precipitate most iron species. The precipitates were removed from the waste water samples by centrifugation for 5 min at 5000 rpm. After filtration, 1 mL disposed water sample was thoroughly mixed with 2 mL MLPOM methanol solution, and the UV-vis absorption spectra were recorded in 1 min later.

### Safety considerations

As Hg^2+^ and most of tested metal ions are highly toxic and have adverse effects on human health, all experiments involving heavy metal ions should be performed under protection. The waste solutions or reagents containing heavy metal ions and the methanol solutions should be collectively reclaimed to avoid polluting the environment.

## Additional Information

**How to cite this article**: Chen, K. *et al.* Label-free colorimetric detection of mercury via Hg^2+^ ions-accelerated structural transformation of nanoscale metal-oxo clusters. *Sci. Rep.* **5**, 16316; doi: 10.1038/srep16316 (2015).

## Supplementary Material

Supplementary Information

## Figures and Tables

**Figure 1:**
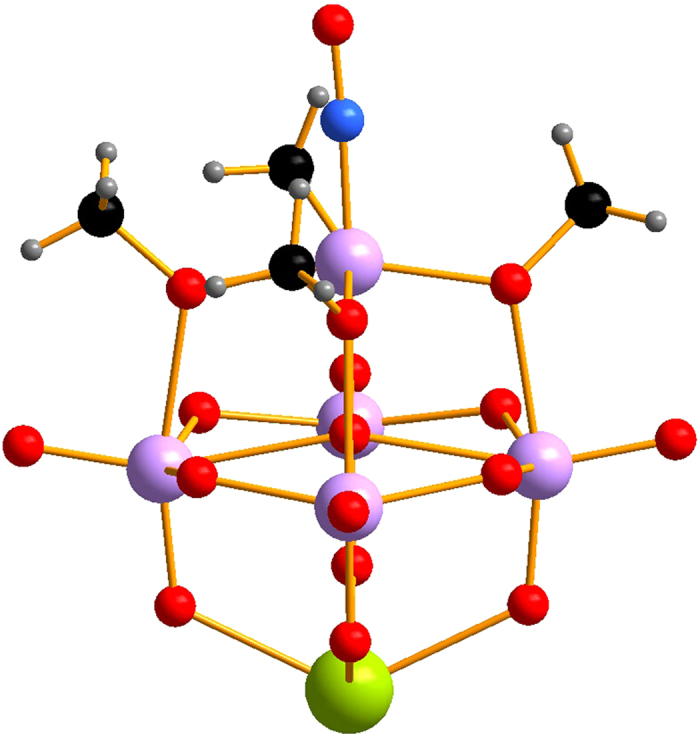
Ball-and-stick representation of MLPOM. Color code: Mo, light purple; Na, lime; O, red; N, blue; C, black; H, light gray.
