# Supplementary material for: Label-free colorimetric detection of mercury via Hg2+ ions-accelerated structural transformation of nanoscale metal-oxo clusters
Source: Sci Rep. 2015 Nov 12;5:16316. doi: 10.1038/srep16316 (PMC4642295; doi:10.1038/srep16316)
Supplement: Supplementary Information [file srep16316-s1.pdf]

Supporting Information

Label-free colorimetric detection of mercury via Hg^{2+} ions-accelerated structural transformation of nanoscale metal-oxo clusters

Kun Chen[†], Shan She[†], Jiangwei Zhang[†], Aruuhan Bayaguud[†] and Yongge Wei*[,†, ‡]

*Correspondence to: yonggewei@mail.tsinghua.edu.cn

[†] Department of Chemistry, Tsinghua University, Beijing 100084, P. R. China.

[‡] State Key Laboratory of Natural and Biomimetic Drugs, Peking University, Beijing 100191, P. R. China.

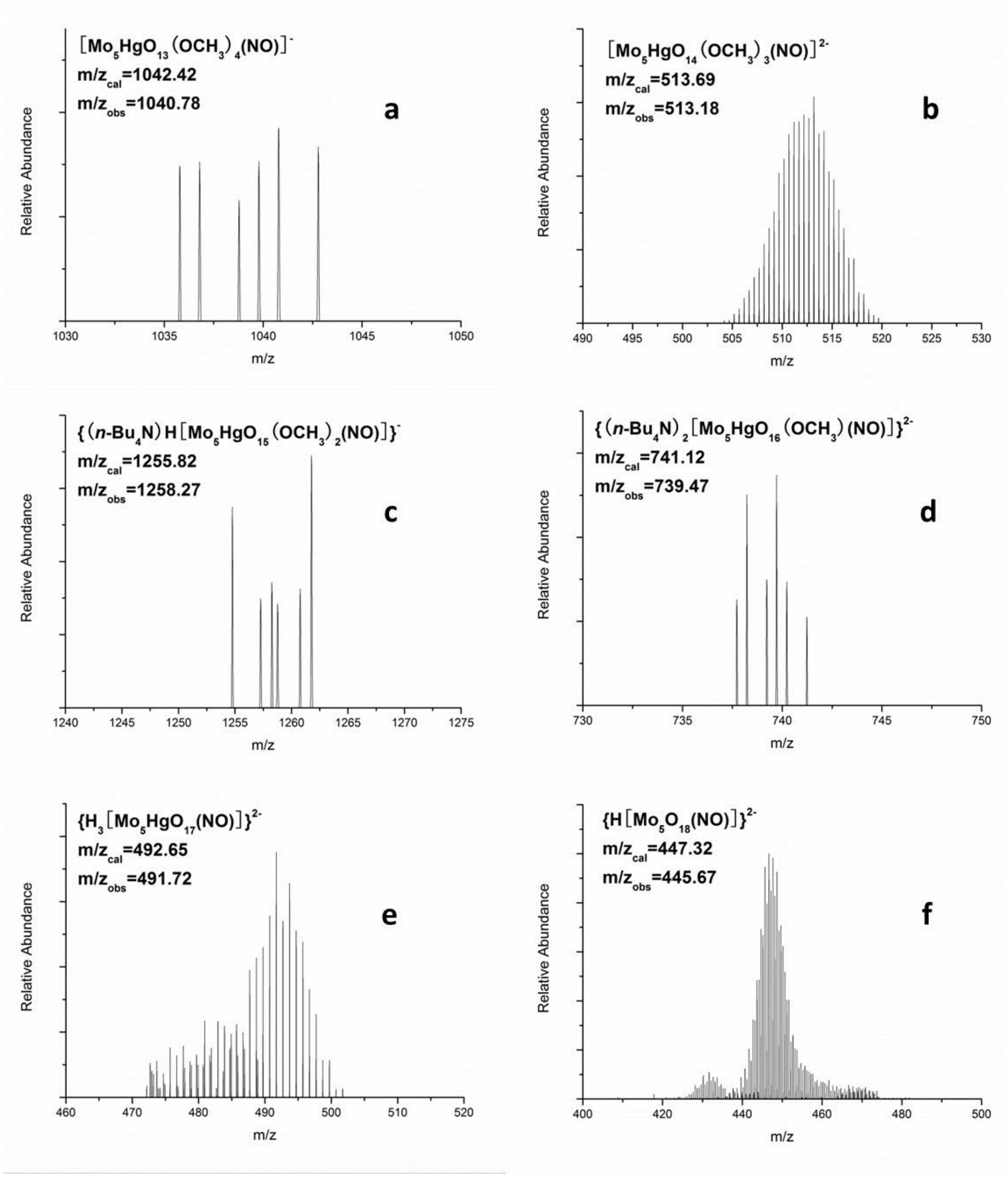

Figure S1. ESI-MS spectra of probably intermediate products: $[Mo_5HgO_{13}(OCH_3)_4(NO)]^-$ (a), $[Mo_5HgO_{14}(OCH_3)_3(NO)]^{2-}$ (b), $[Mo_5HgO_{15}(OCH_3)_2(NO)]^{3-}$ (c), $[Mo_5HgO_{16}(OCH_3)(NO)]^{4-}$ (d), $[Mo_5HgO_{17}(NO)]^{5-}$ (e), and product $[Mo_6O_{18}(NO)]^{3-}$ (f).

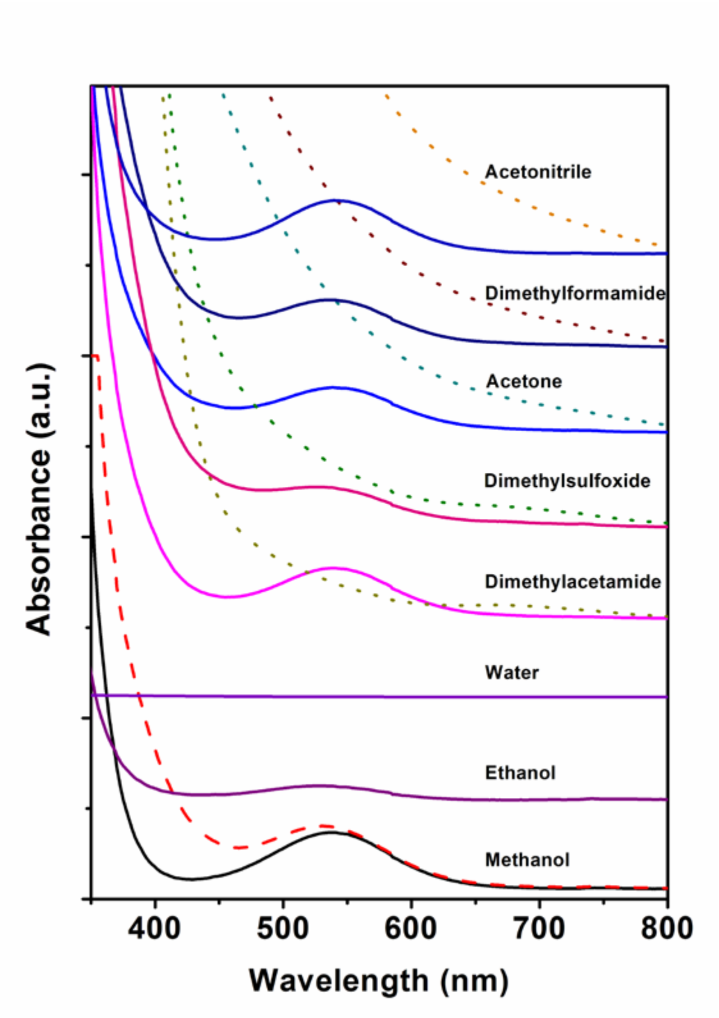

Figure S2. UV-vis spectra of MLPOM in different solvent. Absorbance of MLPOM in methanol (in the presence (red dash line) or absence (black line) of Hg^{2+}), water, ethanol, and other organic solvent: dimethylacetamide, dimethylsulfoxide, acetone, dimethylformamide and acetonitrile (recorded immediately (full line) and 3d later (dot line)).

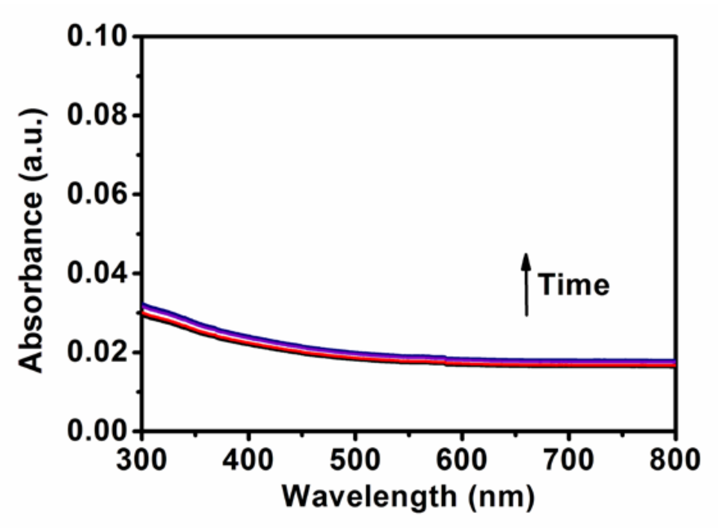

Figure S3. UV-vis spectra of MLPOM in water at different time point. From the bottom up: 10 min (blank), 30 min (red), 60 min (purple), 120 min (navy). Low absorbance is due to the low solubility of MLPOM in water.

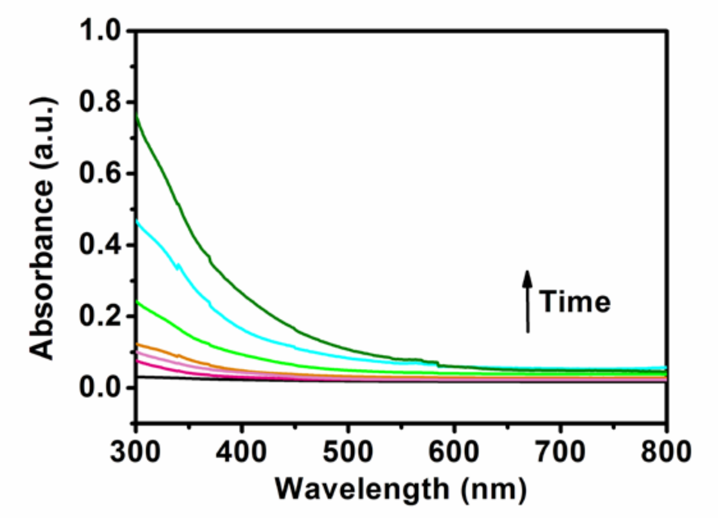

Figure S4. UV-vis spectra of MLPOM in water at different time piont under heating of 80 °C. From the bottom up: 0 min (blank), 5 min (red), 10 min (magenta), 15 min (orange), 20 min (green), 25 min (cyan), 30 min (olive).

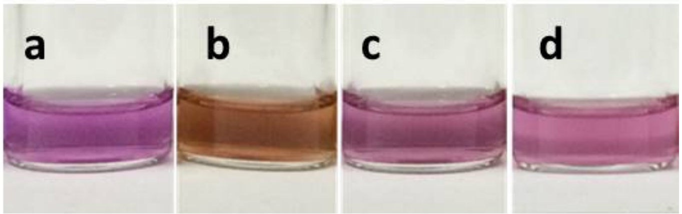

Figure S5. Photographs of MLPOM solution (a), in the presence of Hg^{2+} (b), after adding hydroxylamine hydrochloride (c), and $[Mo_6O_{18}(NO)]^{3-}$ after re-dispersing in methanol (d).

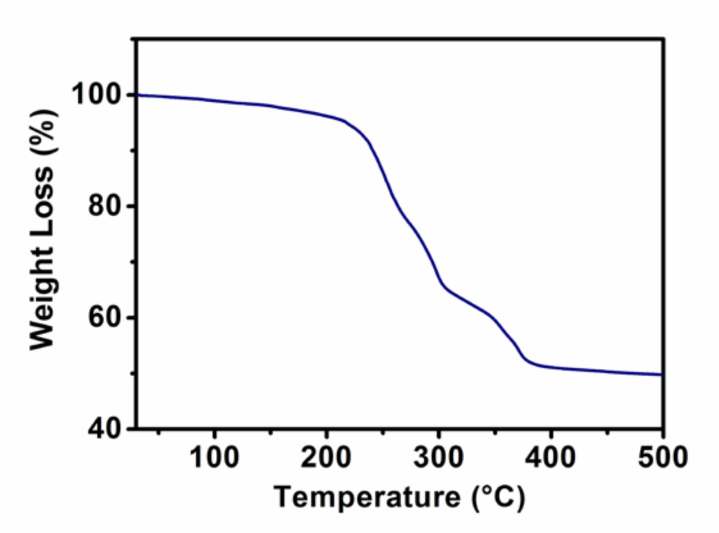

Figure S6. Thermo gravimetric (TG) curve of (n-$Bu_4N)_2[Mo_5NaO_{13}(OCH_3)_4(NO)]$ at a heating rate of 10 °C/min under Ar atmosphere.

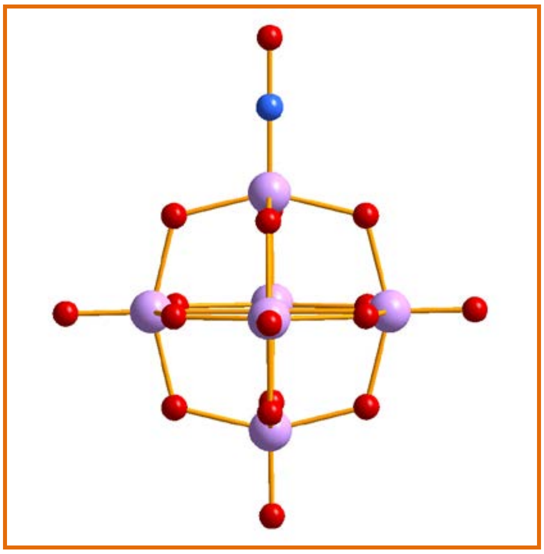

Figure S7. Ball-and-stick representation of $[Mo_6O_{18}(NO)]^{3-}$ anion: Mo, light purple; O, red; N, blue.

References

1. Gouzerh, P., Jeannin, Y., Proust, A., Robert, F. Two novel polyoxomolybdates containing the $(MoNO)^{3+}$ unit: $[Mo_5Na(NO)O_{13}(OCH_3)_4]^{2-}$ and $[Mo_6(NO)O_{18}]^{3-}$. *Angew. Chem., Int. Ed.* **28**, 1363 (1989).

2. Proust, A., Gouzerh, P., Robert, F. Molybdenum oxo nitrosyl complexes. 1. Defect Lindqvist compounds of the type $[Mo_5O_{13}(OR)_4(NO)]^{3-}$ (R = CH_3, C_2H_5). Solid-state interactions with alkali-metal cations. *Inorg. Chem.* **32**, 5291 (1993).
